# Lumbar V3 interneurons provide direct excitatory synaptic input onto thoracic sympathetic preganglionic neurons, linking locomotor, and autonomic spinal systems

**DOI:** 10.3389/fncir.2023.1235181

**Published:** 2023-08-28

**Authors:** Camila Chacon, Chioma V. Nwachukwu, Narjes Shahsavani, Kristine C. Cowley, Jeremy W. Chopek

**Affiliations:** Spinal Cord Research Centre, Department of Physiology and Pathophysiology, Rady Faculty of Health Sciences, University of Manitoba, Winnipeg, MB, Canada

**Keywords:** spinal interneurons, sympathetic preganglionic neurons, motor systems, optical stimulation, propriospinal neurons

## Abstract

Although sympathetic autonomic systems are activated in parallel with locomotion, the neural mechanisms mediating this coordination are incompletely understood. Sympathetic preganglionic neurons (SPNs), primarily located in the intermediate laminae of thoracic and upper lumbar segments (T1-L2), increase activation of tissues and organs that provide homeostatic and metabolic support during movement and exercise. Recent evidence suggests integration between locomotor and autonomic nuclei occurs within the brainstem, initiating both descending locomotor and sympathetic activation commands. However, both locomotor and sympathetic autonomic spinal systems can be activated independent of supraspinal input, in part due to a distributed network involving propriospinal neurons. Whether an intraspinal mechanism exists to coordinate activation of these systems is unknown. We hypothesized that ascending spinal neurons located in the lumbar region provide synaptic input to thoracic SPNs. Here, we demonstrate that synaptic contacts from locomotor-related V3 interneurons (INs) are present in all thoracic laminae. Injection of an anterograde tracer into lumbar segments demonstrated that 8–20% of glutamatergic input onto SPNs originated from lumbar V3 INs and displayed a somatotopographical organization of synaptic input. Whole cell patch clamp recording in SPNs demonstrated prolonged depolarizations or action potentials in response to optical activation of either lumbar V3 INs in spinal cord preparations or in response to optical activation of V3 terminals in thoracic slice preparations. This work demonstrates a direct intraspinal connection between lumbar locomotor and thoracic sympathetic networks and suggests communication between motor and autonomic systems may be a general function of the spinal cord.

## Introduction

Spinal cord injury (SCI) is a life-altering event, resulting in loss of sensation and motor paralysis. In addition, persons living with SCI face a lifetime of impaired automatic (autonomic) bodily functions that affect every aspect of daily living. These impairments include reduced temperature and blood pressure (BP) regulation capability, and reduced or absent ability to activate sympathetic nervous system responses needed during movement and exercise [e.g., increased heart rate (HR), activation of adrenal glands] (Guttmann et al., 1958; Eriksson et al., 1988; Bhambhani, 2002; Ogawa et al., 2014; Cowley, 2018). Recently, spinal electrical stimulation has emerged as a powerful therapeutic to improve motor function after SCI, even when applied years after injury. An unanticipated set of observations that emerged from electrical stimulation trials targeting lower limb motor centers [lumbar spinal cord (SC)], was that it could also improve many autonomic body functions mediated by thoracic spinal sympathetic neurons, including improved blood pressure (BP) and temperature regulation, whole body metabolism and even peak upper body exercise performance [reviewed by Flett et al. (2022)]. The underlying neural mechanisms contributing to these functional gains are unknown. Thus, our interest was to identify whether any neural mechanisms exist that could coordinate locomotor and sympathetic autonomic activity at the intraspinal level.

Normally, autonomic sympathetic preganglionic neurons (SPNs) of the thoracic SC are controlled by autonomic neurons in the rostroventral lateral medulla (RVLM) of the brainstem which has been proposed as a key integration site and descending command center for cardiovascular control (Loewy and Spyer, 1990; Schreihofer and Sved, 2011), thermoregulation (Blessing et al., 1999; Morrison et al., 1999; Bartness et al., 2010a,b) and regulation of lipolysis from white adipose tissues (Bartness et al., 2014). There is considerable anatomical and electrophysiological evidence supporting the RVLM as a key integration center for each of these metabolic and homeostatic functions separately and there is also evidence suggesting that the same or overlapping subsets of brainstem and SPNs may provide neural input to multiple tissues simultaneously, including white adipose tissue and muscle or white adipose tissue and the adrenal glands, etc., (Strack et al., 1988; Loewy and Spyer, 1990; Smith et al., 1998; Kerman et al., 2003; Kerman, 2008; Llewellyn-Smith and Verberne, 2011; Adler et al., 2012; Cowley, 2018). This region of the RVLM is also the final relay for descending locomotor command signals elicited by stimulation of the MLR (Jordan et al., 2008). Indeed, two direct pathways from the MLR were recently identified, one projecting to glutamatergic neurons in the medulla that initiate movement, and one projecting to the cardio-respiratory center located in the RVLM (Koba et al., 2022). It is also of interest to note that chemogenetic activation of serotonin neurons in the parapyramidal region in a genetic rat model elicits hindlimb locomotion and concomitantly increases BP which precedes and outlasts each bout of locomotion, suggesting similar neural mechanisms can activate these two systems simultaneously at the level of the brainstem [(Armstrong et al., 2017); c.f. Figure 4B in Cowley (2018)].

However, given the absence of descending command signals in “complete” SCI, clinical observations of improved sympathetic autonomic functions suggests that intraspinal, ascending neural connections between lumbar locomotor and thoracic sympathetic neural circuitry become activated during lumbar electrical stimulation. We have previously demonstrated it was possible for propriospinal neurons (neurons with dendrites, cell bodies and axons located entirely within the spinal cord) to relay descending locomotor commands from the brain (Cowley et al., 2008, 2010). Further, during fictive locomotion, the lumbar spinal cord is capable of entraining both thoracic (Le Gal et al., 2016) and cervical (Juvin et al., 2012) ventral root activity via propriospinal neurons. However, whether propriospinal relays can integrate information between lumbar locomotor-related spinal neurons and sympathetic autonomic spinal neurons has not been investigated and the underlying intraspinal neural mechanisms mediating these clinical improvements are unknown. We recently developed a conceptual framework to better understand the neural mechanisms integrating locomotor and sympathetic autonomic functions (Cowley, 2018). The model proposes integration between locomotor and sympathetic metabolic and homeostatic neural circuitry occurs at the level of the brainstem, and that activation of SPNs may be mediated in concert with activation of locomotor-related neurons within spinal locomotor pattern generator (CPG) circuitry as part of an integrated spinal locomotor-sympathetic network. We hypothesized that a component of the coordinated increase in sympathetic output may occur through intraspinal projections to sympathetic nuclei that are activated in parallel with lumbar locomotor-related interneurons [c.f. Figure 4 in Flett et al. (2022), (Cowley, 2018)].

In order to examine whether an anatomical and functional propriospinal pathway exists to coordinate activity in locomotor-related and autonomic sympathetic neural circuitry, we chose to focus on one of the 11 cardinal classes of genetically identified spinal neurons (Jessell, 2000). We focused on the V3 population of spinal neurons because the V3 population of interneurons (INs) are important for stabilizing the frequency of locomotion (Zhang et al., 2008), are excitatory, glutamatergic and project both ipsilaterally and contralaterally (Zhang et al., 2008; Blacklaws et al., 2015; Chopek et al., 2018). Computational modeling suggests that V3 INs promote left-right synchrony (i.e., gallop) during high-speed locomotion and have ascending projections from lumbar to cervical regions, providing an anatomical basis for this coordination (Rybak et al., 2015; Danner et al., 2016, 2017, 2019; Zhang et al., 2022). Here we demonstrate a direct intraspinal neural connection between spinal locomotor related V3 neurons in the lumbar spinal cord and thoracic SPNs. Preliminary results were presented previously in abstract and thesis form (Chacon, 2022; Nwachukwu et al., 2022).

## Materials and methods

### Animals

Experimental protocols used complied with the guidelines set by the Canadian Council on Animal Care and were approved by the University of Manitoba animal ethics committee. A total of *n* = 9 adult mice contributed to our anatomical observations. Anatomical and immunohistochemical observations of V3 soma and nerve terminals (*n* = 5) and BDA injections (*n* = 4) were performed in adult Sim1^Cre/+^;Rosa26^floxstopTdTom/+^ (Sim1TdTom; Zhang et al., 2008) mice of both sexes (*n* = 2 of each sex). Adult female and male Sim1^Cre/+^;Rosa26^floxstopTdTom/+^ mice were crossed with Ai32 mice [Gt(Rosa)26floxstopH134R/EYFP/+, Jackson Laboratory, Stock No. 012569] to generate Sim1^Cre/+^; Rosa26^floxstopTdTom/+/Gt(Rosa)26floxstopH134R/EYFP/+^ (Sim1TdTom/Ai32 or Sim1TdTom/ChR2). *In vitro* optogenetic electrophysiological experiments were performed in 23 neonatal (P1-P16) Sim1TdTom/ChR2 mice of both sexes. All electrophysiological experiments were performed in neonates, and the numbers for slice or whole cord preparations are described below.

### BDA injections

#### Recovery surgery protocol for anterograde dye injections

Four adult mice (2 male, 2 female) were subjected to spinal injection of BDA at spinal segments L2 (*n* = 2), L3 or L4/L5 for anterograde tracing. All laminectomies and spinal injections were performed aseptically under anesthesia induced by 5% isoflurane and maintained with 3% isoflurane. Briefly, the skin overlying the vertebrae of interest was opened with a scalpel, back muscles were blunt dissected to reveal the posterior spinous processes and forceps used to perform a single vertebrae laminectomy to expose the spinal cord. Once the dura was opened with a 27-gauge needle, 1 μL of 1% biotin dextran amine (BDA-10,000 mW, Thermo Fisher Scientific Cat# D1956, RRID:AB_2307337) was injected into the exposed spinal tissue. BDA was injected near the left midline using a stereotaxic micromanipulator and at a depth of 700–900 μm from the dorsal surface of the spinal cord. Injection was delivered using a motorized pico-injector with a 75RN SYR 5 μl Hamilton syringe fitted with a single-barrel borosilicate capillary glass (A-M Systems Inc.) pulled to a ∼120 μm tip. The dye was injected over a 5-min period, the needle remained in place for an additional 10 min to prevent dye withdrawal back into the pipette. Each animal received slow-release buprenorphine (0.5 mg/kg), glucose solution (0.02 ml/g), was placed in a heated cage for 2 h until fully functional and then returned to their home-cage where health was monitored daily. Seven days later, animals were terminally perfused and processed as described in “Immunohistochemistry and Imaging.”

### Immunohistochemistry and imaging

#### Immunohistochemistry protocol

Under inhalant isoflurane anesthesia, adult Sim1TdTom mice were transcardially perfused with 4% PFA in phosphate-buffered saline (PBS). Spinal cords were dissected free and placed in 4% PFA overnight, and subsequently cryoprotected in 30% sucrose. For sectioning, the thoracic spinal cord was divided into two approximately equivalent-sized portions, with the rostral portion containing ∼T1–T6/7 and the caudal portion ∼T7–T13. Thoracic spinal cord was sectioned serially in the coronal plane using a cryostat and mounted on glass slides for immunohistochemistry.

To *characterize V3 neuron somas*, thoracic and lumbar spinal cord was sectioned serially in the coronal plane at 30 μm using a cryostat and mounted on glass slides. Since TdTomato is endogenously expressed, sections were processed with 1 × 10 min wash in 0.2% triton-phosphate buffered saline (PBS-T) and then 2 × 15 min washes in 50 mM Tris-hydrochloride (Tris–HCl) and then coverslipped. Images were obtained on a Zeiss Axioscope 40 and sections examined and somas counted at ∼ 450 μm intervals throughout thoracic and lumbar regions *n* = 5 mice. This corresponds with ∼2–3 sections per spinal segment.

To *characterize V3 terminal distribution, SPNs and VGluT2 expression, thoracic spinal cord* was sectioned serially in the coronal plane at 18 μm sections and mounted on glass slides. All sections were washed three times for 10 min each in 0.2% PBS-T, followed by 10-min antigen retrieval (55°C incubation in Tris-EDTA buffer) and an additional three washes in 0.2% PBS-T for 10 min each. A 10% normal donkey serum block in 0.2% PBS-T was applied for 1 h. Sections were then incubated with primary goat anti-dsRED (1:1000, Takara Bio, Cat# 632496, RRID:AB_10013483), goat anti-ChAT (1:400, Millipore, Cat# AB114P, RRID:AB_2313845) and guinea-pig anti-VGluT2 (1:500, Millipore, Cat# AB2251-I, RRID:AB_2665454) for 72 h at 4°C. Sections were then washed three times for 10 min each in 0.2% PBS-T followed by incubation with appropriate secondary antibodies conjugated to Alexa 488, Cy3 and Alexa 647 (1:500; Molecular Probes, Cat# A-11055, RRID:AB_2534102; Jackson Immuno Research Labs, Cat# 711-165-152, RRID:AB_2307443; Jackson Immuno Research Labs, Cat# 706-605-148, RRID:AB_2340476, respectively) for 2 h. Sections were then washed one time for 10 min in 0.2% PBS-T followed by two washes for 10 min each in Tris–HCl 50 mM, then dried, mounted in Vectashield hardset mounting medium (Vector Laboratories) and cover slipped.

To *examine presumed V3 IN terminal density in thoracic IML*, one half of thoracic gray matter was tile-imaged using a 20× objective lens on the Zeiss Airyscan LSM 880 confocal microscope (2–3 Z-stack images through a focusing range of 2.49–4.99 μm) per thoracic section (∼12 images, taken at regularly spaced intervals, corresponded with each thoracic spinal region, *n* = 3 mice). Images were then reconstructed and analyzed in IMARIS. “Spots” detection method was used to filter and select presumed V3 IN terminals. Cell bodies and long neuron processes were excluded from quantification in IMARIS using its size and shape exclusion and re-iterative learning features. A region of interest (ROI) set in IMARIS around the IML (278 × 277 μm) was used to analyze and quantify V3 neuron synaptic terminal density within each spinal tissue section. Individual X–Y coordinates for each synaptic terminal were exported to Excel and GraphPad Prism for subsequent analysis. XY coordinates for each synaptic terminal were also exported and transformed into contour plots using RAWGraphs.^1^

*To determine if V3 terminal apposing thoracic SPNs arose from lumbar neurons* mice injected with BDA were serially sectioned at 18 μm, and processed as described above but immunolabeling for VGluT2 was removed and the secondary antibody Streptavidin647 (1:500, Thermo Fisher Scientific, Cat# S-21374, RRID:AB_2336066) was used at the appropriate step to label for BDA. For BDA-injected mice, every fifth section was examined, representing inter-section distances of ∼ 90 μm throughout the thoracic spinal cord.

#### Imaging V3 terminals apposing thoracic SPNs

A Zeiss Airyscan LSM 880 confocal microscope was used to image VGluT2/TdTom^+^ or BDA/TdTom^+^ neuronal processes apposing SPNs (ChAT^+^ neurons) in thoracic IML. For each rostral (∼T1 – T6/7) and caudal (∼T6/7 – T13) region, 14–20 ROIs containing the IML, with at least 90 μm of inter-section distance, were selected to obtain a representative rostro-caudal distribution of IML regions and SPNs per mouse (*n* = 4). Each IML area was imaged (7–14 Z-stack images through a focusing range of 3.6–12.8 μm) at 63× magnification. Laser settings were optimized and replicated within each animal, for consistency between images. Left and right IML SPNs were selected and imaged based on visibility of at least one SPN soma per ROI. Z-stack images were then imported into Imaris software for 3D reconstruction and quantitative analysis using the “Surface” tool. Synaptic boutons (VGluT2) less than 1 μm from SPN somas were quantified and considered doubled labeled if there was overlap with TdTom^+^ surfaces. Similarly, doubled labeled contacts of BDA/TdTom^+^ were considered as synaptic boutons when less than 1 μm from SPN soma.

#### Verification of BDA injection site

Lumbar tissue was serially sectioned at 30 μm in the coronal plane. Sections were examined and imaged using a Zeiss Axio Imager Z.2 upright microscope to confirm BDA injection sites. Sections were examined visually to identify those with BDA label. The sections with the greatest dye spread were then imaged and injection sites reconstructed using tiled images obtained under 10× magnification within Zen Blue software (Version 3.5). These images were used to identify cytoarchitectural landmarks and confirm rostro-caudal segmental location of injections and transverse distribution of injected dye. Visual inspection of images indicated that 8 or fewer serial sections contained BDA, indicating that the rostro-caudal dye spread was less than one spinal segment.

### Electrophysiology

#### Spinal cord slice preparations

Experiments were performed on spinal sections isolated from 23 neonatal (P1-P16) Sim1^Cre/+^;Rosa26^floxstopTdTom/+^/ Gt(Rosa)26^floxstopH134R/EYFP/+^ (referred to as Sim1TdTom/ChR2) mice. Isolation and preparation of sections, and electrophysiological recording methods have been previously described (Chopek et al., 2018). Briefly, animals were anesthetized with isoflurane, decapitated at the medulla-spinal cord junction and spinal cords dissected out in ice-cold dissecting artificial cerebrospinal fluid (aCSF), composed of (mM): KCl (3.5), NaHCO_3_ (35), KH_2_PO_4_ (1.2), MgSO_4_ (1.3), CaCl_2_ (1.2), glucose (10), sucrose (212.5), MgCl_2_ (2.2), and equilibrated to pH 7.4 with 95% 0_2_ and 5% C0_2_. Once dissected free, thoracic spinal cords were immediately secured in agarose and sectioned at 350 μm using a vibratome (Leica VT1200S, Leica) and then incubated in warm (30°C) aCSF for a minimum of 30 min before performing electrophysiological experiments. Incubation and recording aCSF was composed of (mM): NaCl (111.0), KCl (3.085), D-glucose (10.99), NaHCO_3_ (25.0), MgSO_4_.7H_2_O (0.31), CaCl_2_ (2.52), KH_2_PO_4_ (1.1), equilibrated to pH 7.4 with 95% O_2_ and 5% CO_2_.

#### Intact spinal cord preparations

Spinal cords were dissected free from neonatal Sim1TdTom/ChR2 mice as described above in “Slice preparations” but remained longitudinally intact with dorsal and ventral roots attached. Once free, connective tissue was carefully removed from spinal cord tissue and secured ventral side up with fine insect pins. The retrograde tracer tetramethyl rhodamine (TMR) was diluted with aCSF into a 20% stock solution. Suction pipettes with internal diameters of 100–120 μm were used to draw up a small volume of diluted TMR, and then used to suction ventral roots between T6 and T8 on one or both sides of the spinal cord, depending on ventral root viability and length. Ventral roots were cut close to their exit from the spinal cord immediately before suctioning to facilitate dye uptake and to minimize labeling time. Retrograde labeling of thoracic SPNs continued in the dark at room temperature for at least 3 h (Szokol et al., 2008). A block of agar was prepared in advance with one side of the block cut with a scalpel to provide a 30-degree incline. The spinal cord was then mounted on the agar block and fixed in place with acrylic glue to expose the spinal segment containing TMR-labeled SPNS (and motoneurons). The spinal cord at the level of the agar block was then sectioned with a vibratome. This portion of the spinal cord was then glued to a sylgard-coated (Sylgard, Dow Corning, MI, USA) recording chamber designed and 3D printed in-house specifically for these experiments. In particular, the obliquely cut surface of the spinal cord was placed on a sylgard “ramp” to enable visualization and patch-clamp recordings of SPNs under fluorescence while preserving and maintaining continuity with the lumbar spinal region for optical stimulation. Clusters of labeled SPNs were visualized based on their lateral location and position relative to the central canal in each section. TMR-labeled motoneurons were also labeled in these sections and were clearly distinguishable from SPNs based on their size and location.

#### Whole cell patch-clamp recordings and optogenetic stimulation

Slices or spinal cord preparations were transferred to a recording chamber mounted on a Zeiss AxioExaminer microscope and perfused with oxygenated room-temperature aCSF. Cells were visualized using a 20× wide aperture (1.2 nA) water-immersion objective lens, a CCD camera (CoolSNAP EX OCD Camera, Photometrics, AZ) and Slidebook 6.0 software (Intelligent Imaging Innovations, Denver, CO, USA, RRID:SCR_014300). Patch pipettes were pulled with a P-97 Sutter puller and those with 4–6 MΩ resistances were filled with the following (mM): K-gluconate (128); NaCl (4); CaCl_2_ (0.0001); Hepes (10), glucose (1); Mg-ATP (5); and GTP-Li (0.3). Whole cell patch-clamp recordings were made under current-clamp using a Multiclamp 700B amplifier (Molecular Devices, San Jose, CA, USA, RRID:SCR_014300). Recordings were low pass filtered at 10 kHz and acquired at 25 kHz with CED Power 1401 AD board and displayed and recorded using Signal software (Cambridge Electronic Design, Cambridge UK). Before performing an optical stimulation protocol, rheobase (defined as the minimum current to elicit a single AP was collected to determine cell excitability). Before the optical stimulation protocol, we recorded rheobase and repetitive firing in response to 1 s depolarizing current steps from each SPN. In slice preparations, presumed SPNs (small neurons visualized in the IML, with TdTom fluorescence noted near the soma) were patched and responses recorded. Using a spatial light modulator system as previously described (Chopek et al., 2018, 2021), a region of interest slightly greater than the SPN soma was created for optical stimulation of presumed V3 terminals apposing the patched SPN. Blue light was delivered in five 500 ms pulses at 5 Hz at a laser power of ∼2.5 mW. For intact cord preparations with thoracic surface exposed, SPNs that were retrogradely labeled with RDA were patched and responses recorded. A region of interest covering the ventral L2 segment was created for optical stimulation of presumed ventral L2 V3 neurons and likely axons of passage from caudal lumbar segments. Blue light was delivered in five 500 ms pulses at 5 Hz at a laser power of ∼2.5 mW. In a subset of slice experiments, lumbar V3 neurons were patched and optically activated to confirm that optical stimulation resulted in AP generation in V3 neurons.

### Statistics

#### Statistical analysis

Data are presented as means ± SD. As is common for discovery experiments, no statistical method to predetermine sample size and no randomization or blinding procedures were used. Statistical comparisons using *t*-tests or one-way ANOVA with Tukey’s multiple comparisons tests were performed using GraphPad Prism (V9.5 for MacOS, GraphPad Software, San Diego, California USA^2^). Tests for normality were used to select either parametric or non-parametric tests. Statistical significance was set to *p* < 0.05.

## Results

### V3 IN cell bodies located throughout the thoracic spinal cord

**FIGURE 1:**
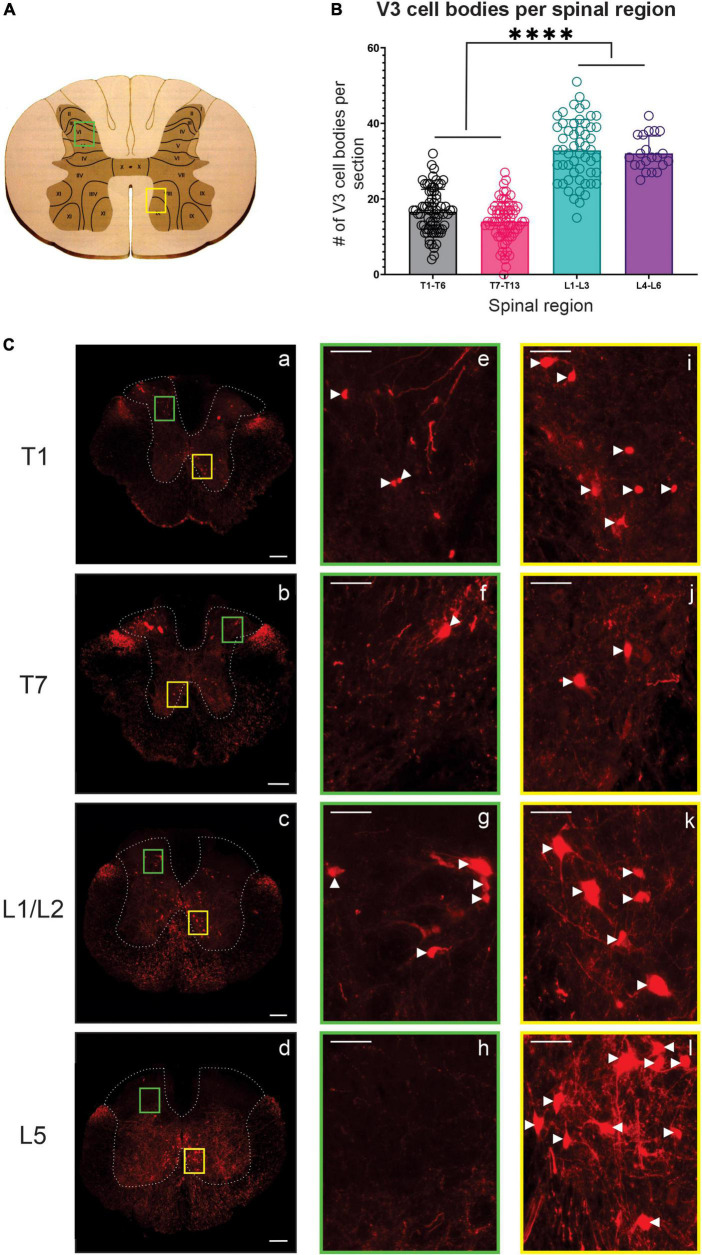
V3 neuronal cell bodies distributed throughout the thoracic and lumbar spinal regions observed in adult mice. **(A)** Schematic and cartoon demonstrating Rexed’s Laminae and regions from dorsal (green boxes) and ventral (yellow boxes) regions for images shown in panel **(C)**. **(B)** Summary data showing mean numbers (±SD) of V3 cell bodies from sections in rostral and caudal thoracic and lumbar regions. Similar numbers of V3 cell bodies were observed within rostral and caudal thoracic and lumbar regions but differed between thoracic and lumbar regions (****P < 0.0001, *n* = 5, one way ANOVA), **(C)** V3 cell bodies were observed at each spinal level in ventral regions (**C**, i–l) and in dorsal regions at T1 (**C**e), T7 (**C**f) and L1/2 (**C**g) but were absent at L5 (**C**h). Scale bar in a–d = 200 μm, and = 50 μm for remaining images. White arrowhead denote V3 soma.

V3 INs cell bodies have been localized within caudal thoracic and upper lumbar spinal regions (Zhang et al., 2008; Borowska et al., 2013) and electrophysiological evidence suggests a role for V3 INs in lumbar locomotor activity [reviewed by Ziskind-Conhaim and Hochman (2017)], but their presence or function(s) in more rostral thoracic regions has not been investigated. Thus, we examined the anatomical distribution of V3 cell bodies throughout the thoracic and lumbar spinal cord (Figure 1). Our findings regarding V3 soma in lumbar regions were consistent with previous reports, with three subpopulations (dorsal, ventral and intermediate subpopulations) in the rostral lumbar and two populations (intermediate and ventral) observed in caudal lumbar regions (e.g., Figure 1Cg vs. Figure 1Ch).

In the thoracic spinal cord, we observed V3 soma throughout T1-T13 at similar dorsal and ventral transverse locations as observed in rostral lumbar regions (Figure 1). V3 soma were not observed within thoracic IML regions. V3 soma in the thoracic region were smaller and less densely distributed compared to V3 cell bodies located in the lumbar region (Compare panels for T1, T7, L1/2, and L5 in Figure 1C – and graph in 1B). On average, similar numbers of V3 cell bodies were observed per 30 μm section in rostral versus caudal thoracic segments (16.6 ± 5.94 V3 soma in T1-T6; 14.0 ± 5.36 V3 in T7-T13; Figures 1B, C T1 vs. Figure 1C T7, *n* = 4). The highest number of V3 somas were observed in lumbar sections (i.e., L1 – L3 with 32.85 ± 8.14 and L4 – L6 with 32.05 ± 4.59 V3 neurons per section, *p* < 0.0001 one-way ANOVA; Figures 1B, C L1/2 vs. Figure 1C L5). In addition to V3 cell bodies, V3 TdTom^+^ fibers were observed throughout the thoracic spinal cord and within IML regions.

**FIGURE 2:**
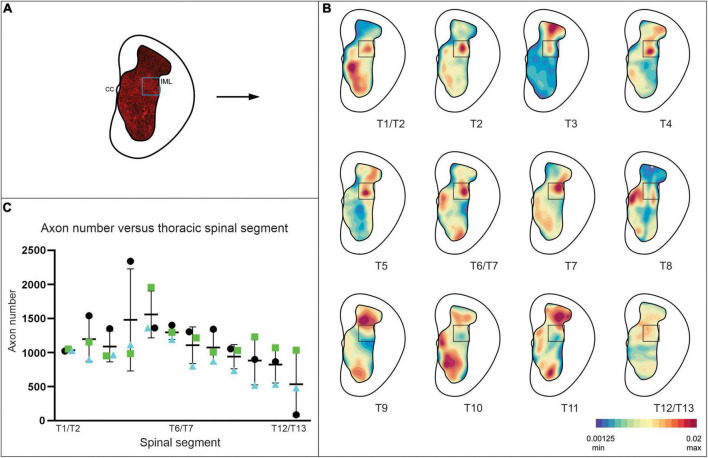
V3 neuron terminals observed within the IML at all thoracic spinal segments, with highest density observed at mid-thoracic levels in adult mice. **(A)** Schematic demonstrating V3 cell bodies and terminals observed in gray matter of spinal cord. XY coordinates of V3 terminals were exported to create contour maps in Imaris. **(B)** Representative contour plots of relative V3 terminal densities within gray matter at each thoracic spinal level from one adult mouse. Note the high density of V3 terminals in the IML and throughout gray matter. **(C)** Graph summarizing numbers of V3 terminals observed in IML ROI (278 μm × 277 μm) at each thoracic spinal level for each animal examined (*n* = 3 animals, 12 sections per animal). Numbers of terminals within the gray matter ranged from ∼2,500 – ∼17,700, and within the IML, terminal numbers ranged from (∼500 – ∼2,400) per section, depending on the rostrocaudal level of the section (*n* = 3).

We therefore characterized the distribution of presumed V3 synaptic terminals within the rostro-causal thoracic gray matter generally and with a particular focus and analysis of terminals within the IML. To do so we identified TdTom^+^ presumed nerve terminals (within sections located at ∼ 900 μm intervals; ∼ 1 per spinal segment) throughout the thoracic spinal cord, including a pre-defined IML region in Rexed’s lamina VII (12 sections per animal, *n* = 3). V3 terminals were identified at all rostro-caudal levels of the thoracic region, within the dorsal and ventral horns, with an expected high density in lamina IX, close to the somatic motor nuclei (e.g., ∼T10 in Figure 2B). For consistency, a pre-defined area (278 μm x 277 μm) of the IML was selected for quantifying numbers of V3 terminals for each section (Schematic in Figure 2A, blue box). Each TdTom^+^ terminal was marked with an XY coordinate and exported from IMARIS to develop contour plots (heat maps) of the relative distribution of terminals throughout gray matter (see representative example of Figure 2A transformed to a “terminals only” map in Figure 2B). Numbers of terminals within the gray matter in each section ranged from ∼2,500 – ∼17,700 (mean = 9,646; 7,577; 5,526 for mouse 1, 2, and 3, respectively) and within the IML, terminal numbers ranged from ∼500 – ∼2,400 per section (mean = 1,279; 1,166; 875 for mouse 1, 2, and 3, respectively), depending on the rostrocaudal level of the section. In particular, there were higher numbers of terminals in the IML region in rostral (∼ >1,000) compared to caudal (∼ < 1,000) thoracic SC (compare heat maps of T1 to T12/13 in Figure 2B and numbers in Figure 2C) with highest numbers observed between ∼T4 and T6 (Figure 2C).

Together these results demonstrate that V3 cell bodies are located throughout the thoracic spinal cord, distributed in similar anatomical populations, but at approximately half the size and number than observed in rostral lumbar regions. These results also demonstrate that there is a large proportion of V3 neuronal terminals within the IML region of the thoracic spinal cord (presumed V3 IML puncta account for 14.3, 15.9, and 21.2% of all puncta within gray matter of one side of spinal cord for animal 1, 2, and 3, respectively).

### 20% of glutamatergic input apposing thoracic SPNs arises from V3 interneurons

**FIGURE 3:**
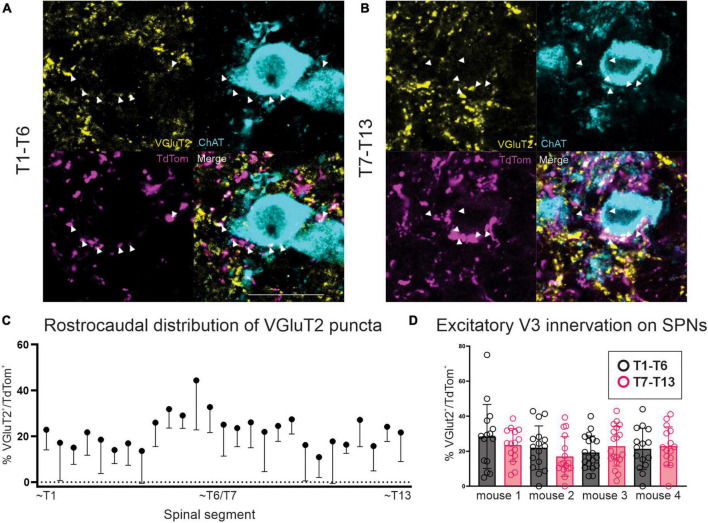
V3 synaptic terminals directly appose thoracic SPNs in adult mice. **(A)** Double labeled VGluT2 and TdTom terminals apposing SPNs in rostral thoracic spinal cord. Double labeled neurons indicated with arrowheads (white in merged image). **(B)** Double labeled VGluT2 and TdTom terminals apposing SPNs in caudal thoracic spinal cord. Double labeled neurons indicated with arrowheads (white in merged image). **(C)** Rostrocaudal distribution of percentage of double labeled VGluT2^+^/TdTom^+^ puncta within thoracic spinal cord. Mean + SD shown. **(D)** Glutamatergic excitatory V3 IN innervation of SPNs averaged ∼20% per section examined, with no significant difference between T1-T6 and T7-T13 regions (*p* > 0.9999 Kruskal–Wallis test, *n* = 4 mice). Scale bar = 20 μm for panels **(A,B)**.

In neurologically intact preparations, VGluT2^+^ terminals constitute the main, if not only, glutamatergic nerve terminal input onto SPNs (Llewellyn-Smith et al., 1992, 2007). In order to determine whether any excitatory V3 terminals observed within thoracic IML directly apposed SPNs, we examined our sections labeled with ChAT (SPNs), VGluT2 and DsRed. Confocal z-stack images from rostral (Figure 3A) and caudal (Figure 3B) thoracic regions were imported into IMARIS software for 3D reconstruction. Using the 3D reconstructions, we calculated and plotted the number of VGluT2^+^ and double-labeled VGluT2^+^/DsRed^+^ synaptic terminals apposing SPNs for sections from rostral and caudal thoracic regions for each animal (Figure 3D). A total of 56–80 SPN sections were reconstructed and analyzed for each rostral and caudal thoracic region per mouse (*n* = 4). On average, 107.2 VGluT2^+^ contacts apposed each SPN (range: 0 – 645 VGluT2^+^ contacts). Over all sections examined for each animal, 16.9–26.6% (*n* = 4 mice) of VGluT2^+^ synaptic terminals were double labeled with DsRed, indicating that these terminals arose from V3 neurons (Figures 3A, B). Similar proportions of excitatory V3 synaptic terminals were observed apposing SPNs within rostral versus caudal thoracic regions in each animal (Figures 3C, D; *p* > 0.9999, *n* = 4, Kruskal–Wallis test). We noted that SPN size varied throughout the thoracic spinal cord, therefore we analyzed the number of V3 terminals apposing each SPN, normalized to observed SPN surface area, and found a similar ratio between rostral (0.63 ± 0.52) and caudal (0.64 ± 0.63) thoracic SPNs suggesting similar densities of V3 terminals apposing thoracic SPNs (*p* = 0.5) regardless of rostrocaudal location or when normalized for relative size of SPN cell body.

### Approximately half of all glutamatergic input apposing thoracic SPNs arises from lumbar V3 interneurons

**FIGURE 4:**
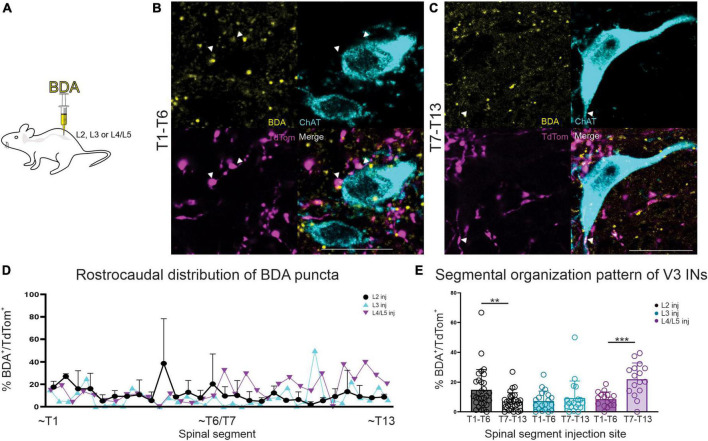
BDA injection in lumbar SC demonstrates ascending V3 neuronal projections to thoracic SPNs in adult mice. **(A)** Schematic of BDA injections. **(B)** Double-labeled BDA and TdTom terminal apposing SPNs in read rostral and caudal thoracic spinal cord (arrowheads and white in merged image). **(C)** Rostro-caudal distribution of BDA^+^ puncta shown by thoracic segmental level, based on lumbar injection level (note that data for *n* = 2 mice injected at L2 is mean + SD). **(D,E)** In animals with BDA injection at L2, higher percentage of BDA^+^/TdTom^+^ terminals were present in sections from rostral thoracic compared to caudal thoracic regions (**p = 0.0067, *n* = 2 mice, Mann–Whitney). In contrast, BDA injections in L4/5 demonstrated higher percentages of BDA^+^/TdTom^+^ terminals in sections from caudal thoracic compared to rostral thoracic regions (***p = 0.0002, *n* = 1, Mann–Whitney). L3 BDA injections showed no significant difference in percentage of BDA^+^/TdTom^+^ terminals in sections from either rostral or caudal thoracic regions (*p* = 0.8568, *n* = 1, Mann–Whitney). Scale bar = 20 μm for panels **(A,B)**.

In order to determine if any of the V3 synaptic terminals apposing thoracic SPNs originated from lumbar V3 neurons, we injected BDA into lumbar spinal cord and then quantified the proportion of V3 contacts (DsRed^+^) that were also BDA^+^ and apposing ChAT^+^ SPNs. Four animals contributed to this series (2 injected at L2, 1 at L3 and 1 at L4/L5). A typical example is shown in Figure 4 with images taken from the rostral (Figure 4B) and caudal (Figure 4C) thoracic regions, demonstrating that a portion of V3 synaptic input onto SPNs originates from V3 neurons in the lumbar region (animal injected at L2). Overall, for each section examined, 3.8–21.9% of total V3 contacts (DsRed^+^) were also BDA^+^. These findings demonstrate that lumbar V3 neurons provide ascending synaptic input onto thoracic SPNs, and double-labeled terminals were observed when injected at either L2, L3, or L4/5 (Figures 4D, E). Overall, 9.7% (+5.1%) of V3 neurons were also labeled with BDA for the entire rostral thoracic region (T1–T6; *n* = 4; range = 5.8–17.8%) and 9.8% (+ 7.8%) for the entire caudal thoracic region (T7-T13; *n* = 4; range = 3.8–21.9%). Thus, approximately half of all V3 contacts on SPNs arose from V3 neurons in lumbar SC.

In this series we also compared the proportion of BDA^+^ and DsRed^+^ presumed terminals in rostral versus caudal thoracic regions to determine if there was any evidence to suggest a somatotopic organization to the ascending input onto thoracic SPNs from lumbar V3 neurons. We compared the proportion of double-labeled terminals observed in rostral (T1-T6; Figure 4B) versus caudal (T7-13; Figure 4C) thoracic regions for BDA injections at rostral lumbar (L2) versus caudal lumbar (L4/5; Figures 4D, E) sites. Figure 4E demonstrates there was a greater proportion of BDA^+^ and TdTom^+^ terminals in sections from rostral thoracic versus caudal thoracic regions for animals injected with BDA at rostral lumbar sites (13.9 vs. 6.4%, respectively) and see also animal injected at L2 in Figures 4B, C (**p = 0.0067, mean values, *n* = 2 mice, Mann–Whitney). Conversely, animals injected with BDA at caudal lumbar sites demonstrated a greater proportion of BDA^+^ and TdTom^+^ terminals in sections from caudal compared to rostral thoracic regions (22.0 vs. 8.5%, respectively, Figure 4E, *n* = 1 mouse, ***p = 0.0002, Mann–Whitney). In contrast, a similar proportion of BDA^+^ and DsRed^+^ terminals were observed in sections from rostral and caudal regions in animals with BDA injection at a mid-lumbar (L3) level (6.95 vs. 9.23%, respectively, Figure 4E, *n* = 1 mouse, *p* = 0.8568 Mann–Whitney). These findings suggest that ascending input from lumbar V3 neurons is somatotopically arranged, as opposed to a pattern of ascending input throughout the entire thoracic region, regardless of V3 lumbar segmental location. Given the small numbers of animals used, it will be of interest to systematically examine the question of rostro-caudal distribution of V3 terminals in a larger sample of more rigorously designed experiments.

### Spinal V2a and V0 neurons do not appose SPNs

As part of our investigations to identify potential sources of intraspinal locomotor-related neuronal input onto sympathetic-related neural circuitry, we performed anatomical experiments to determine whether two other genetically defined candidate spinal neuron populations provide synaptic input to thoracic SPNs or the IML region generally. The first population were the locally projecting, glutamatergic V2a neurons involved in left-right coordination and defined by the transcription factor Chx10 (Al-Mosawie et al., 2007; Lundfald et al., 2007; Crone et al., 2008, 2009). Examined in 2 Chx10:eGFP mice, we observed VGluT2^+^/GFP^+^ fibers within the IML but did not observe any double-labeled (VGluT2^+^/GFP^+^) terminal apposing thoracic SPNs. In these same animals, we did observe VGluT2^+^/GFP^+^ terminals apposing lumbar motoneurons, indicating that our negative findings regarding thoracic SPNs was not due to a weak fluorescent signal or lack of GFP expression in terminals of Chx10 mice.

In a separate series of experiments in Dbx1Cre:tdTomato mice, we examined whether V0 population neurons provide synaptic input onto SPNs. V0 neurons can be subdivided into 3 subpopulations (V0_c_, V0_D_, V0_v_) each with a different neurotransmitter content, projection patterns and roles in locomotion (Moran-Rivard et al., 2001; Pierani et al., 2001; Lanuza et al., 2004; Zagoraiou et al., 2009; Talpalar et al., 2013). Thus, in 2 Dbx1Cre:tdTomato mice, in which all three populations are visualized with red fluorescent protein, we did not observe any TdTom^+^ terminals apposing thoracic SPNs. Similar to our observations for the V2a population noted above, we did observe terminals apposing lumbar motoneurons, indicating that our negative findings regarding thoracic SPNs for the V0 population were not due to a weak fluorescent signal or lack of TdTomato expression in terminals of Dbx1 mice.

### Lumbar and thoracic V3 neurons provide functional excitation to thoracic SPNs

**FIGURE 5:**
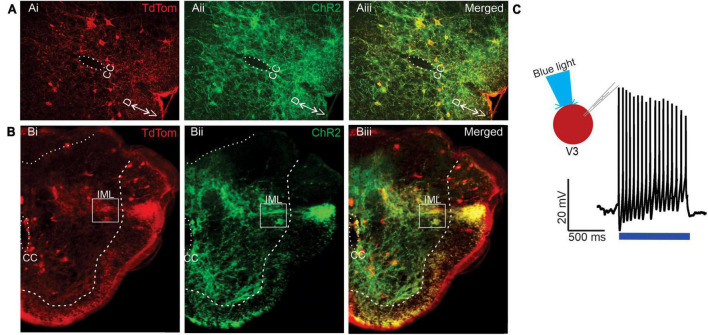
Channel rhodopsin expression in lumbar V3 neurons and nerve terminals in the IML in neonatal mice. **(A)** Transverse sections from L2 spinal cord demonstrating V3 neurons (TdTomato Ai, red) also express channel rhodopsin (Aii, green), with merged image in Aiii, yellow. Dorsal (D) and ventral (V) and central canal (CC) shown. **(B)** Transverse sections of T6 spinal cord demonstrate expression of Tdtomato (Bi) in V3 fibers within IML (white box), channel rhodopsin (Bii), and both in merged images (Biii). **(C)** Optical stimulation evokes multiple action potential in whole-cell patch clamped V3 neurons located in thoracic SC. Example from *n* = 10 cells from 6 mice of either sex.

Since our anatomical investigations demonstrated that V3 spinal neurons provide direct synaptic input onto thoracic SPNs, we performed a series of electrophysiological experiments to determine if activation of V3 neurons provide functional excitation of SPNs. To do so, we bred mice to obtain channel rhodopsin expression in V3 INs and used immunohistochemical microscopic examination to confirm channel rhodopsin expression in V3 neurons in Sim1^Cre/+^;Rosa26^floxstopTdTom/+^/Gt(Rosa)26^floxstopH134R/EYFP/+^ mice (Figure 5A) and that TdTom^+^/Chr2^+^ terminals were present in thoracic IML regions (Figure 5B). We then confirmed that we could elicit action potentials (APs) in lumbar V3 neurons in response to optical stimulation (Figure 5C, *n* = 10). In each V3 neuron examined (*n* = 10/10), optical stimulation over the soma of the patched V3 in lumbar SC elicited repetitive firing of the neuron.

**FIGURE 6:**
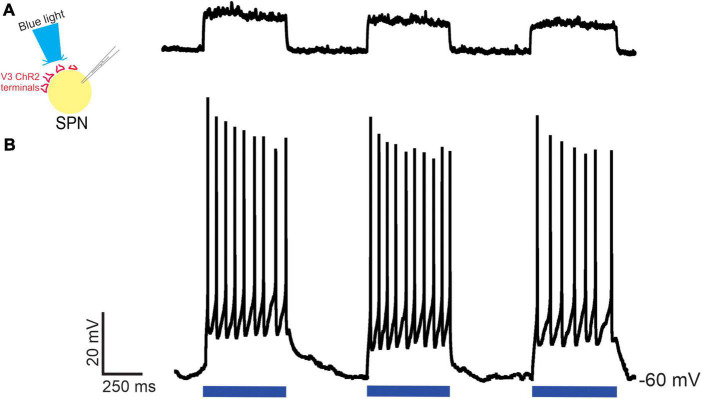
Optical stimulation of V3 nerve terminals elicits excitatory post synaptic responses in thoracic SPNs in neonatal mice. Optical stimulation of V3 nerve terminals could elicit EPSPs [**(A)** *n* = 2] or action potentials [**(B)** *n* = 4] in whole cell patched clamped thoracic SPNs. Examples from *n* = 10 cells from 4 mice of both sexes.

Next, we determined whether V3 neurons provided functional synaptic input to neurons within thoracic IML by testing whether optical stimulation of V3 neuron terminals in thoracic slice preparations could excite presumed SPNs in the IML. Optical stimulation (480 nm wavelength) of V3 terminals directly over the patched presumed SPN using a focal light source from a spatial light modulator elicited excitatory post synaptic potentials (EPSPs, Figure 6A *n* = 2), action potentials (Figure 6B *n* = 4) or no response (*n* = 4, not shown). Thus, in 60% of patched neurons, excitatory responses to optical stimulation were consistently observed for the duration of recording in these presumed SPNs. Presumed SPNs in slice recording had an average rheobase value of 20 ± 5 pA.

**FIGURE 7:**
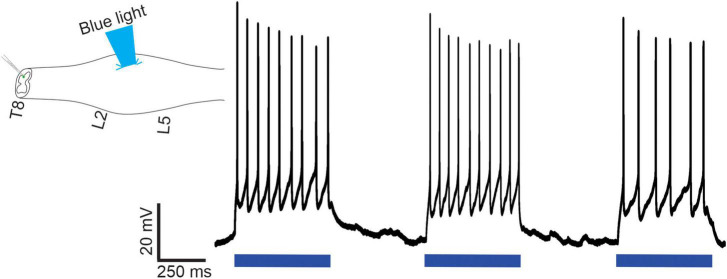
Optical stimulation of lumbar V3 neurons elicits action potentials in thoracic SPNs in neonatal mice. In intact spinal cord preparations, thoracic SPNs labeled with RDA were visualized under fluorescence and whole cell patch clamp recordings were collected in response to optical stimulation of the ventral surface of the L2 spinal segment. In this example, lumbar V3 stimulation consistently evoked action potentials in the patched SPN. Representative example from one of the *n* = 20 cells recorded from 15 mice of either sex.

In our final electrophysiological series, we determined whether optical stimulation of lumbar V3 neurons could excite identified thoracic SPNs located multiple segments rostral to the stimulation site in 15 mice of either sex. As noted in Methods, we targeted SPNs in T6-T8 and used the intact-cord preparation with an obliquely cut surface having a targeted caudal edge at T8. We used TMR applied to related ventral roots to retrogradely label SPNs for visualization while performing patch recordings (*n* = 20 cells in 15 mice). Optical stimulation over the ventral surface of L1-L2 spinal cord elicited EPSPs (*n* = 4), APs (*n* = 5), or failed to elicit a response (*n* = 11) in thoracic SPNs (typical example shown in Figure 7). Backfilled SPNs had a mean rheobase value of 25 ± 10 pA, similar to the presumed SPNs noted above, recorded in slice.

As noted above, 4 of the 10 presumed SPNs and 11 of the 20 identified SPNs did not respond to optical stimulation of either thoracic V3 terminals or lumbar V3 cell bodies, respectively. Given the heterogeneity of target tissues receiving synaptic input from SPNs, one would not necessarily expect all SPNs to receive excitatory input from locomotor-related V3 neurons. Nonetheless, in the 30 presumed or retrogradely labeled SPNs from either slice or intact cord preparations, we noted a relationship between the recorded SPN’s ability to generate repetitive firing in response to current injection and whether optical stimulation elicited an excitatory response. Specifically, of the 15 SPNs that did not produce repetitive firing in response to a 1-s depolarizing current pulse, only 1 showed an excitatory response to optical stimulation. In contrast, 14/15 SPNs that demonstrated repetitive firing in response to current injection also showed an excitatory response to optical stimulation.

Taken together, these anatomical and electrophysiological experiments provide compelling evidence of an ascending intraspinal excitatory neuronal projection from lumbar locomotor-related V3 neurons to thoracic sympathetic preganglionic neurons.

## Discussion

Here, we demonstrate that locomotor related V3 INs project to and excite SPNs throughout the entire thoracic spinal cord and account for ∼20% of glutamatergic input to SPNs. We further demonstrate strong ascending input from lumbar V3 INs to thoracic SPNs (∼ 10% of the VGluT2 input), and, when optically stimulated, evokes post synaptic excitatory responses in SPNs. That is, we demonstrated a functional excitatory intraspinal connection between lumbar V3 INs and thoracic SPNs. We suggest that this newly described intraspinal connection provides an intraspinal capability to integrate locomotor and sympathetic function, which supports the appropriate activation of metabolic and homeostatic support during movement and exercise.

At the supraspinal level, clear evidence demonstrates the brainstem is capable of coordinating and integrating circuitry for movement with related needed sympathetic autonomic functions. Stimulation of the mesencephalic locomotor region (MLR) or subthalamic locomotor region not only results in locomotion but also increases respiratory function (Eldridge et al., 1981) and cardiovascular responses, in an activity and intensity dependent manner (Bedford et al., 1992). Both of these increased sympathetic responses persisted when neuromuscular blockers were applied, suggesting a central source regulating both locomotor and autonomic functions (Chong and Bedford, 1997). Even thinking about moving induces increases in BP and HR in humans temporarily paralyzed by curare, demonstrating similar mechanisms in human and animal models, and suggesting a critical link between motor and sympathetic autonomic systems at supraspinal levels (Gandevia et al., 1993). Confirming that these responses are mediated by a central source, two direct pathways from the MLR were recently identified, one projecting to glutamatergic neurons in the medulla that initiate movement, and one projecting to the cardio-respiratory center located in the RVLM (Koba et al., 2022). Further, opto- or chemogenetic activation of serotonergic neurons in the RVLM induce increases in systemic BP which precedes and outlasts bouts of simultaneously elicited locomotor activity, suggesting an integrative role for the brainstem in coordinating locomotor and autonomic support systems [(Armstrong et al., 2017), c.f. Figure 4B in Cowley (2018)].

Evidence for coordination within brainstem locomotor and autonomic circuitry has been demonstrated for glutamatergic Chx10 neurons located in the gigantocellular nucleus of the medulla. Anatomical and electrophysiological evidence indicates these neurons contribute to the generation and coordination of locomotion (Bretzner and Brownstone, 2013; Bouvier et al., 2015; Schwenkgrub et al., 2020; Usseglio et al., 2020) and when ablated, respiration rate generated in the nearby pre-Botzinger complex is reduced (Crone et al., 2012). Similarly, MLR stimulation increases blood pressure, heart rate and vasoconstriction prior to any rhythmic motor activity, even in the absence of movement in unanesthetized, decerebrate paralyzed rats (Bedford et al., 1992; Chong and Bedford, 1997; Koba et al., 2005). Anatomical studies using pseudorabies virus injection into a variety of motor and sympathetic autonomic tissues demonstrated higher-order supraspinal neurons that function in both somato-motor and sympathetic systems, termed somato-motor-sympathetic neurons (Kerman et al., 2003, 2006, 2007; Kerman, 2008). These findings suggest that communication and adequate activity in both brainstem autonomic and locomotor centers is sufficient and required for movement to occur. Based on evidence provided here, we propose a similar coordinating role between these systems within the spinal cord, and that V3 INs are involved in integrating and communicating between both autonomic SPNs and lumbar locomotor networks. Such integration between somatic motor and sympathetic autonomic systems appears to be a common feature at the afferent-spinal reflex level as well.

For example, Sato and Schmidt (1973) described multiple somatic-motor and cutaneous non-nociceptive afferent evoked intra-segmental and multi-segmental sympathetic metabolic and homeostatic spinal reflexes in multiple species (Schmidt and Weller, 1970; Sato, 1973, 1987; Flett et al., 2022). These reflexes are additional to the well described “pressor effect” of lower limb afferents on BP regulation and include increases and decreases in catecholamine release from adrenal glands in anesthetized preparations, dependent upon direction of mechanical stimulation of fur follicles, such that brushing the fur “against” the grain increases, and brushing the fur “with” the grain decreases adrenal release (Sato and Schmidt, 1973; Loewy and Spyer, 1990; Schreihofer and Sved, 2011), Similar to somatic motor reflexes, these sympathetic metabolic reflexes may become reversed or exaggerated after SCI due to a loss of descending control [(Sato, 1973) and reviewed in Flett et al. (2022)].

Our conceptual framework proposed that the spinal cord has the capability of integrating motor and sympathetic functions, and that this residual function may become critically important after SCI, when descending commands are removed (Cowley, 2018; Flett et al., 2022). Our recent scoping review supports this concept, as spinal electrical stimulation below injury targeted to lumbar regions to improve motor activity could also improve sympathetic autonomic functions, particularly in those with cervical level injury with their severely reduced or absent ability to activate sympathetic autonomic spinal neuronal circuitry (Flett et al., 2022). We hypothesized the mechanisms mediating improved locomotor and autonomic function involved ascending propriospinal neural networks. Ascending propriospinal neurons arising from the lumbar spinal cord can coordinate and entrain the locomotor rhythm on both thoracic respiratory neurons (Le Gal et al., 2016) and the cervical CPG network (Juvin et al., 2012; Zhang et al., 2022). Although we examined multiple glutamatergic neurons (V2a, V0_D_, V0_V_, and V0_C_), including several that are classified as propriospinal, our results indicate that V3 INs provide a major source of spinal glutamatergic input to SPNs. V3 INs are ideal candidates to communicate and integrate locomotor and sympathetic functions as they have long ascending projections from lumbar to cervical spinal cord, and are involved in stability of locomotion and coordination between left and right hindlimbs and between hindlimbs and forelimbs (Zhang et al., 2008, 2022; Blacklaws et al., 2015; Danner et al., 2019), all of which is needed to sustain overground locomotion, particularly at higher intensities or durations.

We demonstrated that ∼20% of all VGluT2^+^ synaptic contacts on SPNs arise from V3 INs. As SPNs primarily receive glutamatergic input from neurons using the glutamatergic transporter VGluT2 (Llewellyn-Smith et al., 2007), this indicates that ∼20% of all glutamatergic inputs to SPNs are from V3 INs. Thus a large portion of excitatory input onto SPNs arises from V3 INs and about half this input appears to originate from lumbar V3 INs. Although it is known that SPNs receive intraspinal synaptic input from neurons using a variety of neurotransmitters including glutamate, GABA, and acetylcholine, little is known of the source of the neurons providing input to SPNs (Llewellyn-Smith, 2009), and our findings represent an important first step in this identification process. It is likely that intraspinal input to SPNs also includes locally projecting spinal interneurons located in laminae V, VII, and X (Llewellyn-Smith, 2009). In our preliminary investigations, we did not observe any input from locally projecting Chx10^+^ spinal INs or from DBX^+^ spinal INs apposing SPNs. Thus, in addition to the ∼20% of glutamatergic input to SPNs from V3 INs, we conjecture that a large portion of remaining glutamatergic excitatory input to SPNs arise from brainstem centers including the RVLM.

Our findings reflect the neural mechanisms and pathways present in the intact preparation and do not account for any plasticity-induced changes that may occur after spinal cord injury. As such, these experiments reveal the presence of an ascending lumbar locomotor to thoracic sympathetic pathway that is anatomically present and functional in the intact preparation, but its relative role during locomotion under normal circumstances is not known. Our data shows these connections are functionally present in neonates and anatomically present in adults. Our conceptual framework suggests these intraspinal ascending connections may play a relatively minor role in the intact system (Cowley, 2018; Flett et al., 2022), but this remains to be tested. After SCI, there may be additional, plasticity-related changes that occur to increase the relative importance of such ascending connections. For example, Hou et al. (2008), described a substantive and widespread increase in propriospinal ascending connections between pelvic afferents and thoracic sympathetic autonomic neurons. Others, such as Chakrabarty and Martin (2011a,b), have shown that there is considerable capacity for functional plastic changes with either development or after SCI within sensory-motor systems at the spinal level, which can be influenced by descending input from the cortex (Smith et al., 2017). Whether the combination of spinal cord injury-induced plasticity in these ascending autonomic to autonomic connections may be additionally facilitated by activating locomotor-related circuitry that in turn, increases these sympathetic autonomic responses, is currently unknown. For those with sub-optimal sympathetic activity after SCI, activation of locomotor circuitry to normalize sympathetic responses may have potential to provide an important means of improving health and life quality after SCI, provided responses can be controlled and maintained within a non-hypertensive response range. They may also provide a spinal target for stimulation for increasing exercise capacity and performance after injury.

Although we demonstrated that V3 INs provide a substantial portion of excitatory amino acid input to SPNs, we have not identified the final target tissue V3 IN input to SPNs activates. Like somatic motor systems, the autonomic input to bodily tissues typically follows a somatotopic organization, although an intact connection to T1 is required to activate a variety of sympathetic autonomic tissues including the heart, sweat glands and vasculature smooth muscle (Cowley, 2018; Flett et al., 2022). For instance, the greatest density of nerve terminals and fibers in the IML arising from Enkephalin expressing neurons occurs in T1-T8. For substance P expressing neurons the greatest density occurs between C8-T12 and T11-L1, and for serotonergic input, T1-T5 in cat (Krukoff et al., 1985). A similar distribution of serotonergic fibers within the IML was also seen in the rabbit, with the greatest density in T3-T6 and L3-L4 and minimal numbers T1 and T10-T12 (Jensen et al., 1995). This differential rostro-caudal neuromodulatory input to SPNs, could permit specific functional groups of SPNs to respond in different ways to the same homeostatic challenge. Future experiments should examine the relationship between intraspinal projections and ultimate body targets of SPN output. We know, for example, that rostral thoracic SPNs via Superior Cervical Ganglia and Stellate Ganglion are involved in the regulation of upper body targets such as pupils, salivary glands, sweat glands of the head and arms, and the heart. SPNs in mid thoracic spinal cord via Celiac ganglion control mesenteric vasculature, gut motility and secretion and the adrenal medullae. SPNs in the caudal spinal cord aid in regulation of lower body organs such as urinary bladder and reproductive organs. In addition, there is a general somatotopic organization to sympathetic innervation of sweat glands, vascular smooth muscle and white adipose tissues that typically follows the dermatomes below T1 (Cowley, 2018).

Although preliminary, we observed a peak density of V3 IN contacts within T4-T7 segments, which via post-ganglionic sympathetic neurons innervate the heart and adrenal medulla. In addition, we also demonstrated that V3 INs innervate thoracic somatic MNs. Taken together, it is conceivable that in response to increased motor function, lumbar V3 INs provide additional excitation to SPNs to increase heart rate and stroke volume, increase release of circulating catecholamines from adrenal glands and fatty acid release from white adipose tissue, as well as activate thoracic MNs to meet the increased metabolic demands for respiration and posture during overground locomotion. In addition, one would expect less excitatory input from locomotor-related V3 neurons to SPNs involved in regulating gut motility or reproductive organs as these are not vital for, and may impede, sustained movements or exercise. That is, there is likely a selective innervation of SPNs from neurons involved in locomotion, to ensure adequate organ and tissue support for movements. Consistent with this, our findings showed that ∼ 50% of patched SPNs responded to V3 activation with depolarization or APs. This finding may reflect limited activation of locomotor and sympathetic systems *in vitro* under these experimental conditions or reflect that SPNs in the thoracic region provide neural input to a variety of tissues and organs, many of which are inhibited, or show reduced activation during locomotor activities. One might predict future research will identify ascending locomotor-related neurons that provide inhibitory input to thoracic SPNs as well.

Overall, this is the first demonstration of an anatomical and functional connection within the spinal cord between neurons involved in locomotion with neurons involved in sympathetic functions. We propose that similar to the brainstem, communication between locomotor and autonomic centers also occurs within the spinal cord. Long projecting ascending V3 propriospinal neurons may be a key neuron population ensuring that adequate metabolic and homeostatic resources are maintained during sustained rhythmic activity such as exercise. It will be of interest to determine how activation of lumbar V3 neurons *in vivo* alters whole body metabolic and homeostatic processes at rest and during locomotion. These neurons may also be of therapeutic importance for improving autonomic and motor function after SCI.

## Data availability statement

The raw data supporting the conclusions of this article will be made available by the authors, without undue reservation.

## Ethics statement

The animal study was approved by the University of Manitoba Bannatyne Campus Animal Care Committee. The study was conducted in accordance with the local legislation and institutional requirements.

## Author contributions

KC and JC conceived the research project and edited and revised the manuscript. CC, KC, and JC conceived and designed experiments and interpreted the results. CC, CN, NS, and JC performed experiments. CC and NS analyzed the data. CC and JC prepared figures. CC, CN, JC, and KC drafted the manuscript. All authors approved the final version of the manuscript.

## Footnotes

1
https:app.rawgraphs.io

2
www.graphpad.com
